# Inflammatory diseases causing joint and bone destruction: rheumatoid arthritis and hemophilic arthropathy

**DOI:** 10.1007/s00774-024-01520-8

**Published:** 2024-06-10

**Authors:** Asuka Terashima, Kumiko Ono, Yasunori Omata, Sakae Tanaka, Taku Saito

**Affiliations:** 1 Bone and Cartilage Regenerative Medicine, The University of Tokyo Hospital, 7-3-1 Hongo, Bunkyo-Ku, Tokyo 113-8655 Japan; 2 Department of Joint Surgery, Research Hospital, The Institute of Medical Science, The University of Tokyo, 4-6-1 Shirokanedai, Minato-Ku, Tokyo 108-8639 Japan; 3 Orthopaedic Surgery, Sensory and Motor System Medicine, Graduate School of Medicine, The University of Tokyo, 7-3-1 Hongo, Bunkyo-Ku, Tokyo 113-8655 Japan

**Keywords:** Rheumatoid arthritis, Hemophilia, Hemophilic arthropathy, Joint destruction

## Abstract

Various diseases and conditions cause joint disorders. Osteoarthritis (OA) is characterized by the degeneration of articular cartilage, synovitis, and anabolic changes in surrounding bone tissues. In contrast, rheumatoid arthritis (RA) and hemophilic arthropathy (HA) display marked destruction of bone tissues caused by synovitis. RA is a representative autoimmune disease. The primary tissue of RA pathogenesis is the synovial membrane and involves various immune cells that produce catabolic cytokines and enzymes. Hemophilia is a genetic disorder caused by a deficiency in blood clotting factors. Recurrent intra-articular bleeding leads to chronic synovitis through excessive iron deposition and results in the destruction of affected joints. Although the triggers for these two joint diseases are completely different, many cytokines and enzymes are common in the pathogenesis of both RA and HA. This review focuses on the similarities between joint and bone destruction in RA and HA. The insights may be useful in developing better treatments for hemophilia patients with arthropathy and osteoporosis by leveraging advanced therapeutics for RA.

## Introduction

Various conditions or diseases can impair the function of articular joints, and subsequent joint pain restricts the activity of daily living. The most prevalent joint disorder is osteoarthritis (OA), a degenerative condition caused by aging, obesity, overuse, joint instability, or other factors. In most of OA joints, articular cartilage is degenerated, and symptoms and disease progression are associated with inflammation of the synovial membrane, known as synovitis. In contrast to cartilage, anabolic changes are commonly observed in bone tissues surrounding the affected joints, e.g., osteophyte formation and subchondral bone sclerosis.

Joints are also affected by autoimmune disorder, such as rheumatoid arthritis (RA), systemic lupus erythematosus, ankylosing spondylitis, psoriatic arthritis, juvenile idiopathic arthritis, and scleroderma. Among them, RA is the most prevalent and has been well researched for decades. The pathogenesis of RA is determined by multiple factors, including genetic and environmental backgrounds. The primary tissue of the RA pathogenesis is the synovial membrane. Autoantibodies, such as rheumatoid factor and anti-citrullinated protein antibodies, are often observed in patients with RA. T cells and B cells play crucial roles in the inflammatory response [1]. Chronic synovitis leads to the release of cytokines, such as tumor necrosis factor (TNF) and interleukins (IL), along with other inflammatory mediators [1]. These cytokines further exacerbate synovitis, resulting in the destruction of cartilage and bone. In contrast to anabolic changes of bone tissues of joints with OA, both articular cartilage and bone tissues are usually destructed in RA if not appropriately treated.

The destruction of cartilage and bone following synovitis is also observed in patients with hemophilia. Hemophilia is a rare genetic disorder caused by a deficiency in certain blood clotting factors, particularly Factor VIII (in hemophilia A) or Factor IX (in hemophilia B). These factors are crucial for effective blood clotting in response to injury. Recurrent bleeding into synovial joints destroys articular cartilage and bone tissues, i.e., hemophilic arthropathy (HA). Synovitis plays a pivotal role in HA, as well as in RA. Blood within the joint space triggers inflammatory responses in the synovium, further damaging the articular cartilage and bone tissues [2]. Of note, many aspects of the pathophysiology of HA retain unelucidated since the molecular mechanisms of HA have not been studied as extensively as those of RA. In this review, we describe the similarities and differences in joint and bone destruction between RA and HA, which may contribute to providing better methods for maintaining physical activity in patients with hemophilia by leveraging advanced therapeutics for RA.

## Epidemiology and phenotypes

Rheumatoid arthritis (RA) is a systemic autoimmune disease characterized by severe joint destruction with inflammatory synovitis. The estimated global prevalence of RA is 0.5–1.0%, with geographical differences [1]. The prevalence of RA in Western countries is higher than in Asia [1, 3]. There are an estimated 800,000 RA patients in Japan, with a male-to-female ratio of approximately 1:3 [4]. The prevalence of RA increased with age, typically after 30 years, and onset age peaks in the late 60s [5]. Recently, the number of people developing RA at even older ages has been increasing in Japan. In the older patients, the gender difference in prevalence decreases, with a male–female ratio is 1:2 to 3 [5]. RA patients display pain, stiffness, and swelling of multiple joints caused by synovitis. RA typically manifests symmetrically in the metacarpophalangeal and proximal interphalangeal joints of hands and feet, as well as in the wrist, shoulder, hip, knee, and ankle joints [1], but onset patterns are diverse. Formerly, chronic inflammation led to the destruction of articular cartilage and surrounding bone tissues, resulting in serious joint deformity over time. The elucidation of RA pathophysiology has contributed to the development of effective drug treatments, including methotrexate, biological drugs, and Janus kinase (JAK) inhibitors. These latest therapeutics have markedly improved the prognosis of RA patients, although treatment remains challenging for some sub-population of the patients.

Hemophilia is usually inherited via X chromosome with a mutation of the FVIII or FIX gene [6]. However, both genes are prone to new mutations, and about 30% of all cases result from spontaneous genetic variants, without family history of the disease [6]. Because hemophilia is X-linked, more than 98% of patients with hemophilia are male in Japan [7]. In the United States, the male incidence of hemophilia A and hemophilia B at birth is about 1 per 5000 and 1 per 30,000, respectively [8]. According to a report in 2022, there are 5776 hemophilia A patients and 1294 hemophilia B patients in Japan [7]. The affected neonates and infants often experience spontaneous hemorrhage, such as abnormal bleeding after blood draws, surgery, or trauma, subcutaneous bleeding, and intracranial hemorrhage [8]. The incidence of intra-articular bleeding increases as the patients grow up, and that of joint pain increases in their teens. The intra-articular bleeding typically occurs in the knee, ankle, and elbow [8]. More than half of hemophilia patients find it difficult to squat by the time they reach their 30s, and over 80% of patients aged 60 or older experience some form of joint dysfunction. Therefore, the onset of HA is earlier than that of RA. The development of blood clotting factor products has significantly improved hemostatic control in hemophilia patients. However, the occurrence and progression of HA cannot be completely prevented or inhibited even by these drugs. A magnetic resonance imaging study showed that joint damage was identified in approximately 20% of clinically asymptomatic patients with HA, despite receiving prophylaxis [9].

## Genetic backgrounds

In accordance with the former epidemiologic studies indicating a genetic component in RA, genome-wide association studies have identified at least 100 susceptibility loci for RA [10–13]. These loci are enriched for immune or inflammation-related genes [12]. Certain genes, such as HLA-DR4, HLA-DR1, and HLA-DRB1, have been associated with an increased risk of RA [1]. There is a range of other susceptibility genes for RA: protein tyrosine phosphatase non-receptor type 22 (PTPN22) is involved in regulating immune responses [12, 14]; tumor necrosis factor alpha-induced protein 3 (TNFAIP3) is a regulator of inflammation [15–17]; C–C chemokine receptor type 6 (CCR6) is responsible for the recruitment of immune cells to inflamed tissues [18]; CCR6-expressing Th17 cells are potent inducers of inflammation (they can migrate to the inflamed synovium under the guidance of chemokines, including CCL20, which binds to CCR6 [19]); the interferon regulatory factor 5 (*IRF5*) gene (coding an immune response-related protein) is also associated with other autoimmune diseases, as well as RA [20, 21]. The diversity in genetic backgrounds as described above might be linked to the diversity in the phenotype of RA.

On the other hand, hemophilia is a typical monogenic disorder. Although the severity of hemophilia depends on the type of genetic mutation [22], the genetic factors for HA are much less diverse than those for RA. Genetic studies for hemophilia have mainly focused on the emergence of inhibitors against infused clotting factor concentrates, which make managing hemophilia more difficult [23]. Rather than genetic factors, the amount and mode of exercise, lifestyle factors, and medication compliance may be more deeply involved in the occurrence or progression of HA [24, 25].

## Cytokines and synovitis

The development of effective RA treatments can be attributed to identifying cytokines, their related signaling pathways, and responsible immune cells that contribute to synovitis and produce the cytokines [1]. Among the various cytokines, IL-6, IL-1β, and TNF play pivotal roles. They are deeply involved in inflammatory pathways, particularly the nuclear factor kappa B (NF-κB) and the JAK-signal transducer and activator of transcription 3 (STAT3) pathways. NF-κB is widely associated with acute and chronic inflammatory responses. IL-1β and TNF are representative cytokines that activate NF-κB, which upregulates IL-1β, TNF, and IL-6 [26]. IL-6 activates the JAK-STAT3 pathway, exerting various effects. IL-6 is also a downstream molecule of the JAK-STAT3 pathway. The feedback loops of these cytokines and pathways enhance inflammation in RA [26]. The JAK-STAT3 and NF-κB signaling pathways interact with each other and work collaboratively through IL-6 and other cytokines [26].

Various cells produce IL-6, including macrophages, T cells, endothelial cells and fibroblast-like synoviocytes (FLS) in the RA synovium, and it contributes to sustained synovitis through the signaling pathways described above. The significance of IL-6 is clearly displayed by previous findings that IL-6-deficient mice display little or no antigen-induced arthritis [27]. Indeed, the antibody against IL-6 has shown efficacy in treating RA patients [28]. TNF is another therapeutic target in RA. TNF is produced by macrophages, T cells, and FLS [27, 29]. TNF can boost NF-κB and further enhance IL-6 production. TNF signaling plays a predominant role in establishing an experimental model of RA [30]. TNF inhibitors have also been widely used for treating RA patients with high efficacy [31]. In addition, IL-17 and IL-23 are associated with RA pathogenesis [27]. IL-17 is predominantly secreted by Th17 cells, and IL-23 contributes to the differentiation and maintenance of Th17 cells.

The trigger for HA is intra-articular bleeding. Intra-articular bleeding causes iron deposition in the synovium as a product of hemoglobin degradation [32]. The monocytes/macrophages can phagocytose red blood cells and process the iron from hemoglobin [32]. Recurrent bleeding and excessive iron deposition subsequently enhance catabolic cytokines and enzymes, which could lead to the destruction of articular cartilage and bone tissues [32]. Hemoglobin-derived iron can act as a catalyst for the Fenton reaction in which hydroxyl radicals are formed from hydrogen peroxide, and may directly damage to chondrocytes [33].

Some cytokines in HA are common to RA. In hemophilic arthritis mice, bleeding into the joints can trigger the release of IL-1β, IL-6, C-X-C motif chemokine ligand 1 (CXCL1) and CCL2, contributing to joint inflammation and pain [34]. These cytokines have also been observed in patients with RA [35, 36]. The production of IL-6 was significantly higher in HA synovium than in RA or OA synovium in vitro [37]. Anti IL-6 receptor antibody improved acute joint swelling and pathologic changes in the synovium and cartilage of the experimental model of HA [38]. Blood components elicit the production and release of TNF from macrophages in vitro [39, 40]. TNF expression increased in the soft tissue of mice joints after injury [41] and in the synovial fluid of patients suffering from hemarthrosis caused by an ACL injury [42]. In addition, the activation of osteoclasts and the resulting osteopenia in the trabecular bone adjacent to the hemarthrosis were prevented in TNF-deficient mice [41]. In HA patients, anti-TNF therapy decreased synovitis and hemarthroses [43]. IL-1β is also released in response to joint bleeding. It promotes inflammation, cartilage degradation, and the recruitment of immune cells to the affected joint. IL-1β has been investigated as a potential therapeutic target in HA [44].

## Catabolic enzymes

Matrix metalloproteinases (MMPs) are enzymes that can be produced in response to inflammation and are involved in cartilage degradation. In healthy conditions, MMPs could help to maintain tissue homeostasis by breaking down and remodeling components of the extracellular matrix [45]. This process may be essential for normal tissue repair and turnover. However, in arthritis, MMPs are often overproduced and cause excessive tissue remodeling or damage [35]. They break down the structural proteins within cartilage, such as collagen and proteoglycans, leading to cartilage erosion and bone loss. Furthermore, MMPs could have not only a degradative function in cartilage but also a pro-inflammatory effect [35]. They have the ability to cleave and activate pro-inflammatory cytokines, such as pro-IL-1 and the latent type of transforming growth factor-β (TGF-β) [46, 47]. Although TGF-β is essential for tissue repair, TGF-β is a potent osteoclastogenesis inducer [27].

MMPs are found in the joint fluid of patients with RA and HA [1, 48]. There are more than 20 members of the MMP family. MMP-1 levels are elevated in various arthritic conditions [49, 50]. MMP-3 is one of the most extensively studied MMPs in RA [51]. Elevated levels of serum MMP-3 are associated with synovial inflammation and joint destruction in RA [52]. It can be measured in the synovial fluid of RA patients [52, 53]. MMP-1, -2, and -3 are detected in ligaments of patients with HA more abundantly than in those tissues of OA patients [48]. Like RA, the MMPs are expected to enhance joint destruction in HA. MMP-13, which is involved in the degradation of type II collagen, has also been explored in RA [54]. Joint bleeding in rats significantly increased the expression of MMP-13, compared with a control group [55]. Furthermore, elevated MMP-13 expression has been observed in knee joint punctured-*F8* knockout mice, which mimic hemophilia A [56].

## Bone destruction and systemic decreases in bone mineral density in RA and HA

A key phenomenon common in RA and HA is bone erosion subsequent to synovitis. This requires activation of osteoclasts, the only cells that resorb bone. Important insights into the role of osteoclasts in the pathogenesis of bone erosion around the periarticular lesions were provided by examining experimental arthritis in osteoclast-deficient mice. The receptor activator of NF-kB ligand (RANKL) is a key cytokine that is indispensable for osteoclastogenesis. RANKL knockout mice, which lack the capacity to form osteoclasts, failed to develop significant bone erosions in arthritis models [27]. Moreover, knockout mice of c-fos, an essential transcription factor for osteoclastogenesis, cannot generate osteoclasts [57]. When the *c-fos*^*−/−*^ mice were backcrossed with TNF transgenic mice which spontaneously develop arthritis, bone erosion was not observed despite comparable joint inflammation to their wild-type littermates [58]. These findings provide definitive evidence that osteoclasts are essential for bone destruction in arthritis.

**Fig. 1:**
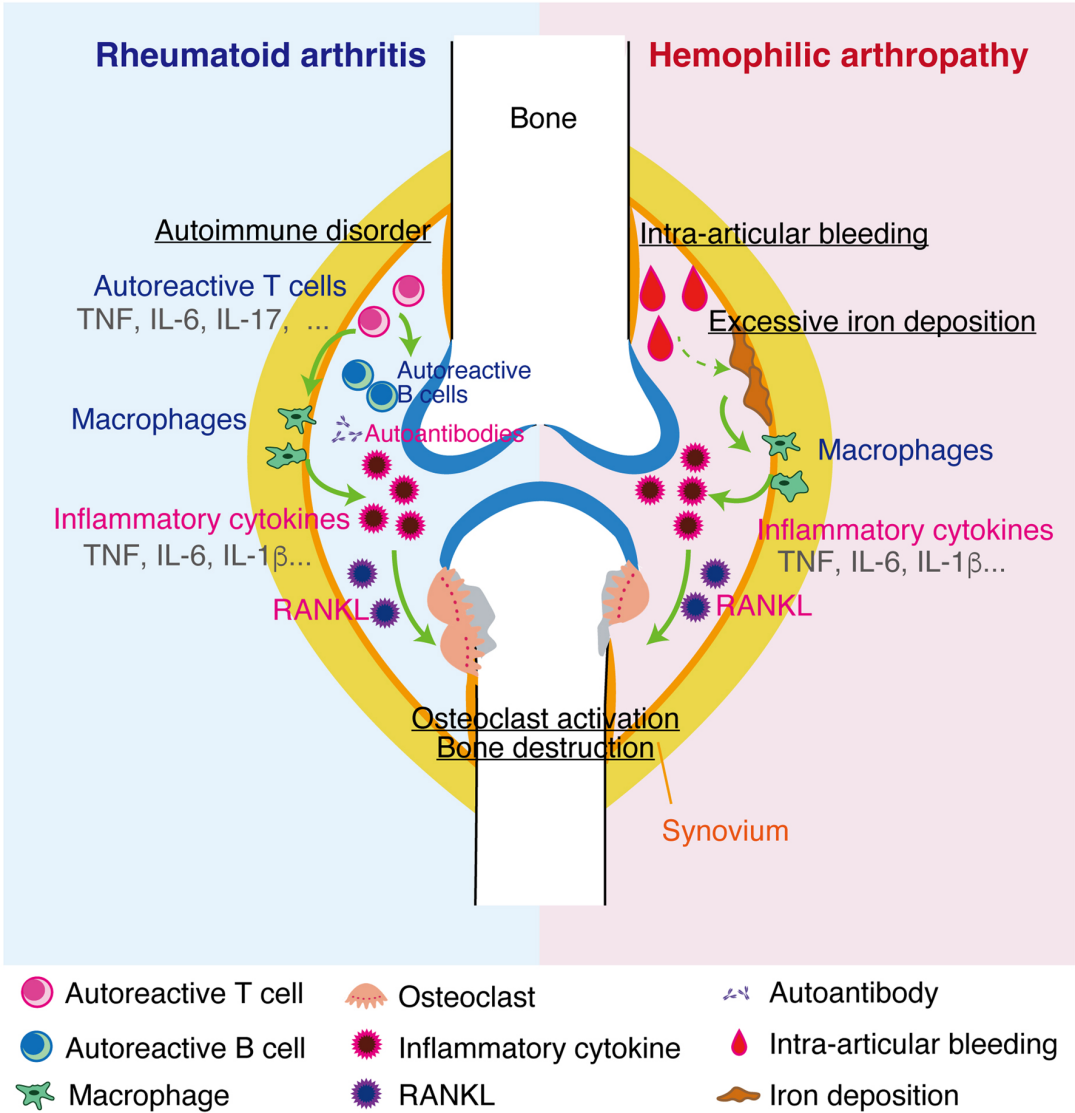
Joint destruction in rheumatoid arthritis (RA) and hemophilic arthropathy (HA). This figure shows the main causes of each pathological condition and the cells and cytokines affected within the joint. This figure shows the main causes of each pathological condition and the cells and cytokines affected within the joint

Notably, the cytokines responsible for RA pathogenesis positively regulate osteoclast formation by increasing RANKL expression in osteoclast-supporting mesenchymal cells [27]. RANKL is induced by IL-6 stimulation in FLS from RA patients, and the IL-6 production is elicited by TNF, IL-1β, and IL-17 [59]. High expression of RANK and RANKL has also been detected in the HA synovium, suggesting osteoclastogenesis is activated in HA [60]. There was no significant difference in osteoclast numbers in the subchondral bone of femoral heads between HA and RA groups [61], nor were there significant differences in the expression of RANKL, RANK, TNF, and IL-1β in femoral heads derived from HA and RA patients [61]. To promote a better understanding of the similarities and differences between RA and HA described above, an outline of this review is illustrated in Table 1 and Fig. 1.

**Table 1:** Comparison between rheumatoid arthritis (RA) and hemophilic arthropathy (HA)

	Rheumatoid arthritis (RA)	Hemophilic arthropathy (HA)	Reference
Estimated number of patients in Japan	Estimated 800,000	Estimated 5000	[1], [7]
Male-to-female ratio	1:3	The majority are male. (Since hemophilia is an X-linked disease, the ratio is unbalanced.)	[4], [7]
Typical age of joint pain onset	Late 60s	Under 30s	[5], [8]
Affected joints	Symmetric arthritis in various joints	Mainly in the elbow, knee, and ankle joints	[1], [8]
Causes	Autoimmune disorder	Intra-articular bleeding caused by coagulation disorders	[1], [8]
Highly associated cytokines	TNF, IL-6, IL-1b, IL-17, IL-23, etc.	IL-6, TNF	[27–31], [38–40]
Highly associated genes	HLA-DRB1, PTPN22, TNFAIP3, TNFAIP3, CCR6, IRF5, etc.	Blood clotting factors (F8, F9, etc.)	[12], [14–21]

RA and HA have similarities and differences. It is thought that the large differences in the causes affect the onset age and site. Similarities can be seen in inflammatory cytokines

Systemic decreases in bone mineral density (BMD), as in osteopenia and osteoporosis, are common in both RA and HA patients, along with periarticular bone destruction. Bone absorption is frequently enhanced in both patient groups [62, 63]. Previous studies suggest that similar pathogenic factors, such as RANKL and inflammatory cytokines mentioned above, are involved in osteoclastogenesis upregulation [62, 63]. The decreased physical activity caused by joint pain and subsequent vitamin D deficiency also reduces BMD in these patients [62, 63]. Glucocorticoids, which have been widely used for treating RA, further exacerbate osteopenia or osteoporosis and increase the risk of fracture [62]. Many studies have shown the efficacy of anti-resorptive agents such as bisphosphonate and denosumab for RA-associated or glucocorticoid-induced osteoporosis [62]. In contrast, there has only been one clinical trial for managing low BMD in patients with hemophilia, indicating the efficacy of ibandronate [63, 64]. Considering the similar pathogenesis underlying systemic decreases in BMD, efficient regulation of disease activity contributes to preventing osteopenia or osteoporosis in both groups of patients. Appropriate medication for osteoporosis should be considered when the risk of fracture rises.

## Conclusion

Although the triggers are quite different, the inflammatory cytokines and some secreted proteins involved in the synovitis are similar between RA and HA. Enhanced bone resorption caused by excessive osteoclastogenesis is also similar in both conditions. Denosumab, anti-RANKL antibody drug, is effective not only for osteoporosis but also for bone erosion in RA joints [65]. Considering the similarities in bone destruction between RA and HA, anti-bone resorptive agents or biologic drugs against IL-6 or TNF may demonstrate efficacy against HA as well as RA. Meanwhile, unlike RA, HA typically occurs early in life, sometimes in childhood. To overcome HA, further research is required to elucidate the underlying molecular mechanisms, which may, in turn, contribute to improving the management of joint disorders, including OA, RA, and HA.
